# Supplementary material for: Regulation of CRE-Dependent Transcriptional Activity in a Mouse Suprachiasmatic Nucleus Cell Line
Source: Int J Mol Sci. 2022 Oct 13;23(20):12226. doi: 10.3390/ijms232012226 (PMC9602552; doi:10.3390/ijms232012226)

Supplements

Supplementary Figure S1A ßIII Tubulin

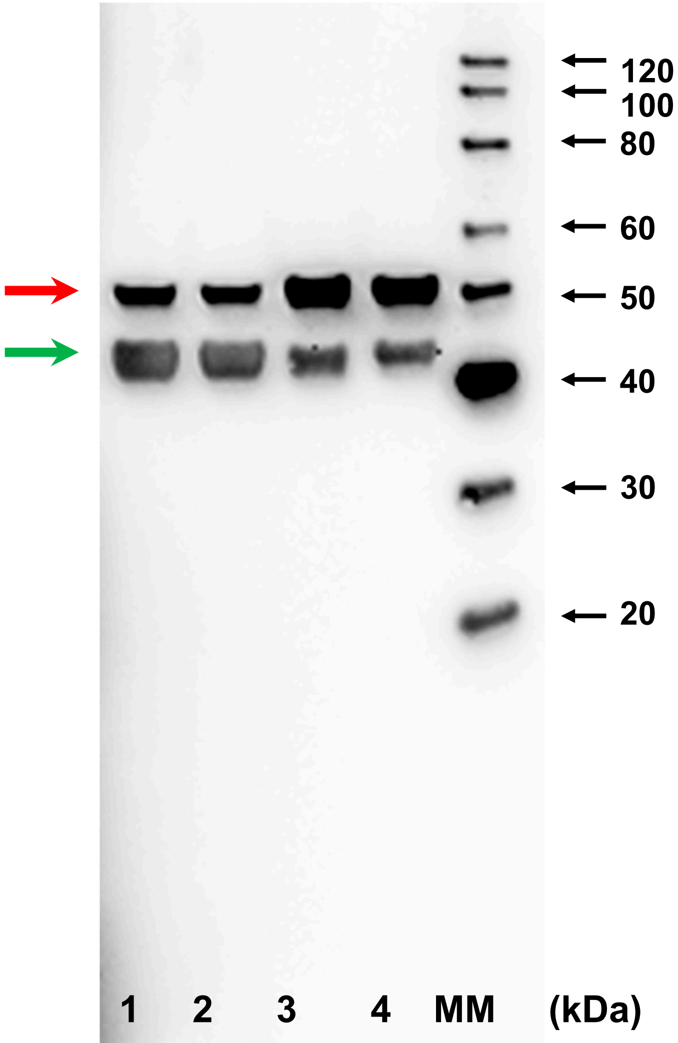

ßIIITubulin/ß-Actin

Supplementary Figure S1B MAP2B

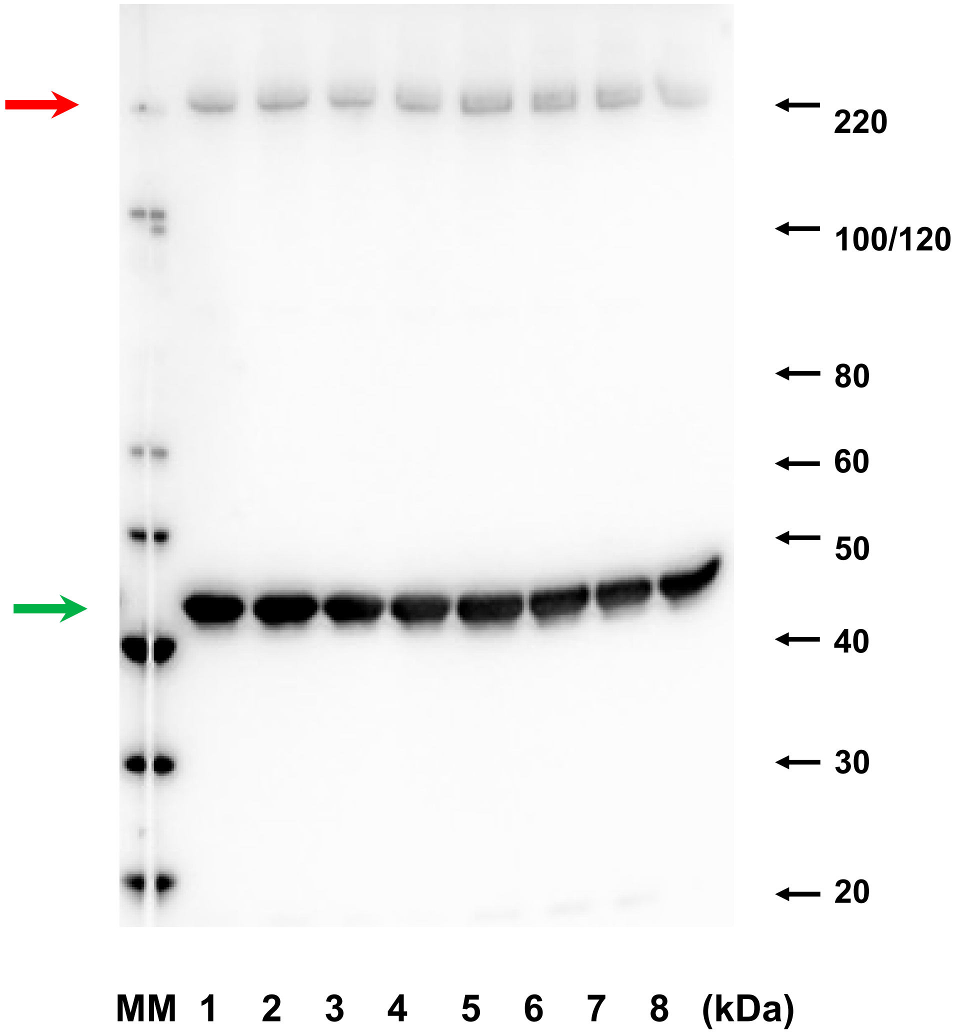

MAP2B/ß-Actin

Supplementary Figure S2

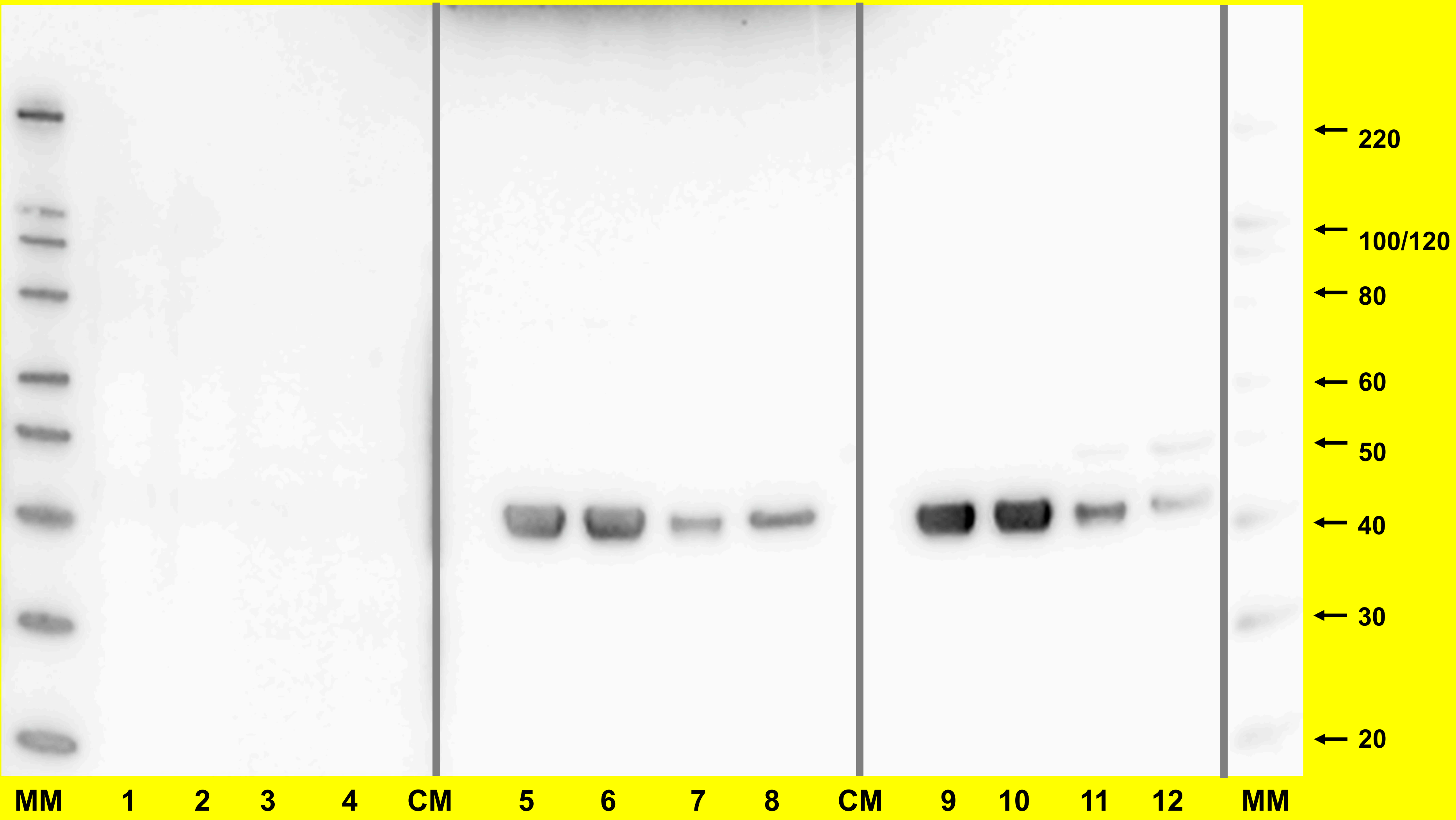

Doublecortin (41 kDa) Nestin (240 kDa)/Actin GFAP (50 kDa)/Actin

Supplementary Figure S3 pCREB antisera comparison

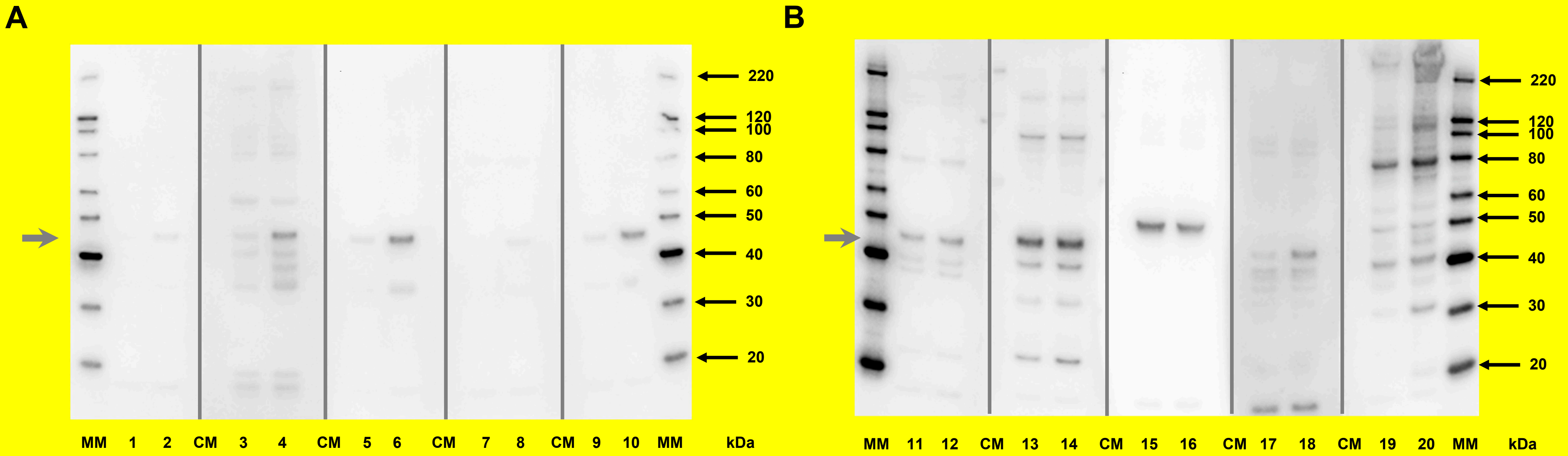

Antigen	Host	Method	Supplier	Dilution		Numbers	
pCREB	Rabbit	Western Blot	Sigma C9102	1:1000		1,2	A
pCREB	Rabbit	Western Blot	SAB 11052	1:1000		3,4	
pCREB	Rabbit	Western Blot	CST#9198s	1:1000		5,6	
pCREB	Rabbit	Western Blot	CST#9191s	1:1000		7,8	
pCREB	Rabbit	Western Blot	87G3; CST	1:1000		9,10	
pCREB	Rabbit	Western Blot	Millipore 06-519	1:1000	Lot:1924367	11,12	B
pCREB	Rabbit	Western Blot	Millipore 06-519	1:1000	Lot:2325090	13,14	
PKA RIIα	Mouse	Western Blot	BD Transduction labs	1:5000		15,16	
pCREB	Rabbit	Western Blot	R&DAF2510	1:1000		17,18	
p-PKA substrate	Rabbit	Western Blot	100G7E; CST	1:2000		19,20	

Supplementary Figure S4 pCREB Western blot vs immunocytochemistry

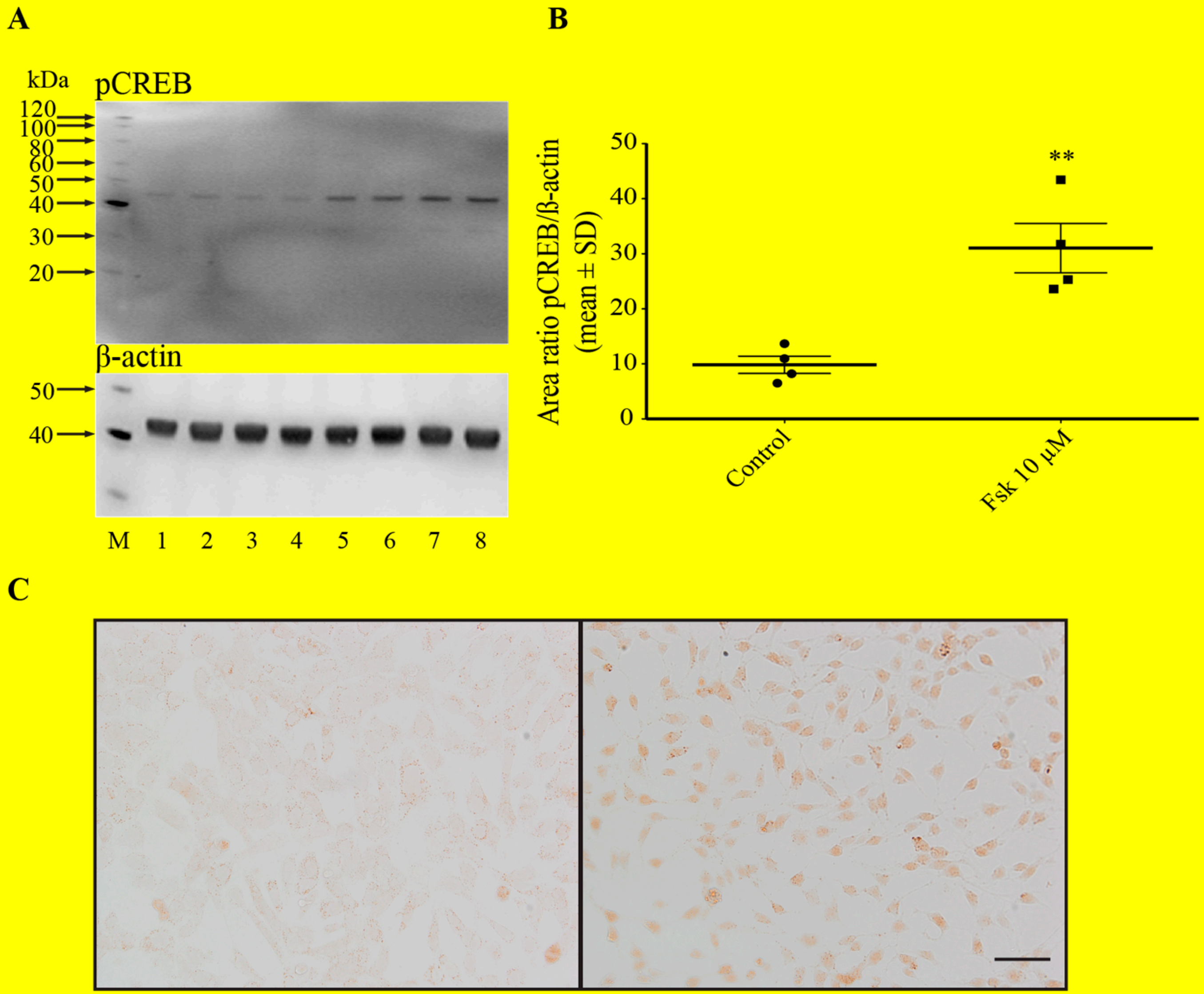

Supplementary Figure S5: proVIP in cells extract (1-4; 9-12) and cell culture supernatant (5-8;13-16)

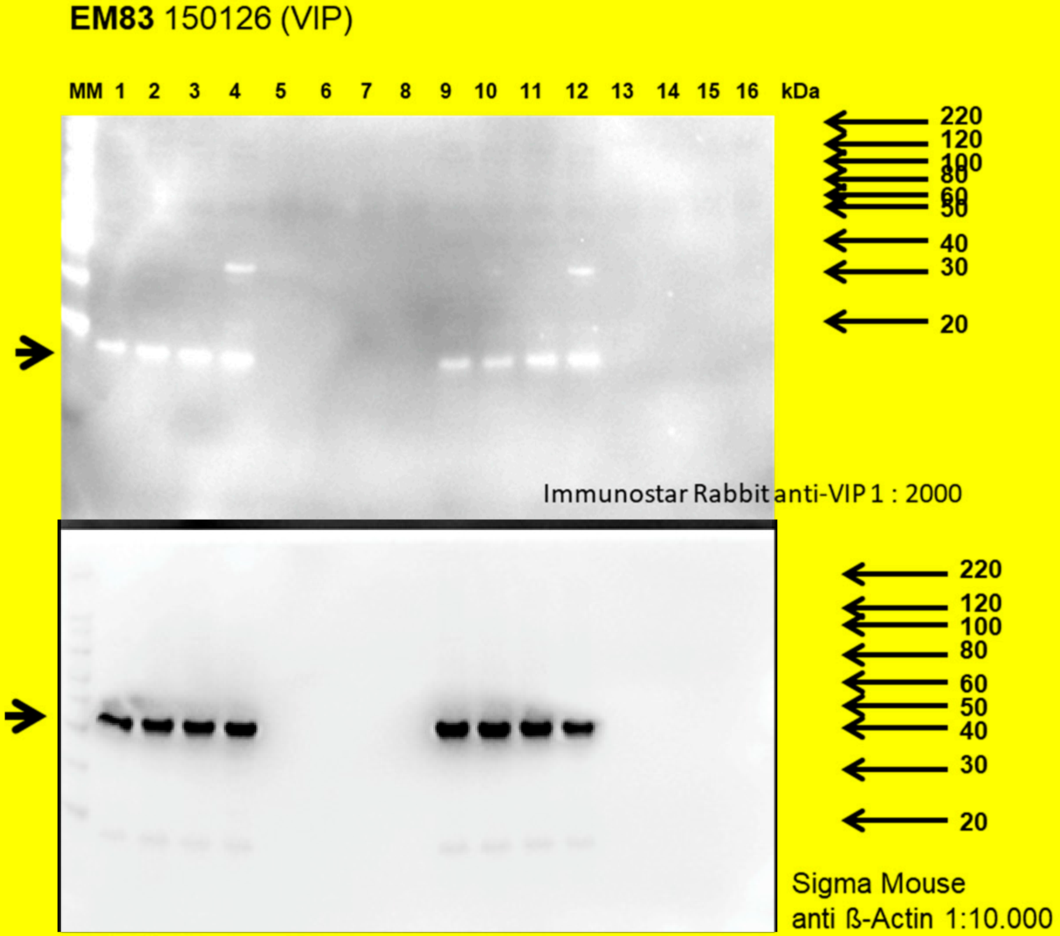

1-4 = SCN CREluc cell extract
5-8 = SCN CREluc supernatant
9-12 = SCN CREluc cell extract
13-16 = SCN CREluc supernatant

MM Magic marker
Sec. AB sc-2054 goat-anti-rabbit 1: 50.000
Sec. AB DAKO goat-anti-mouse 1: 50.000

A

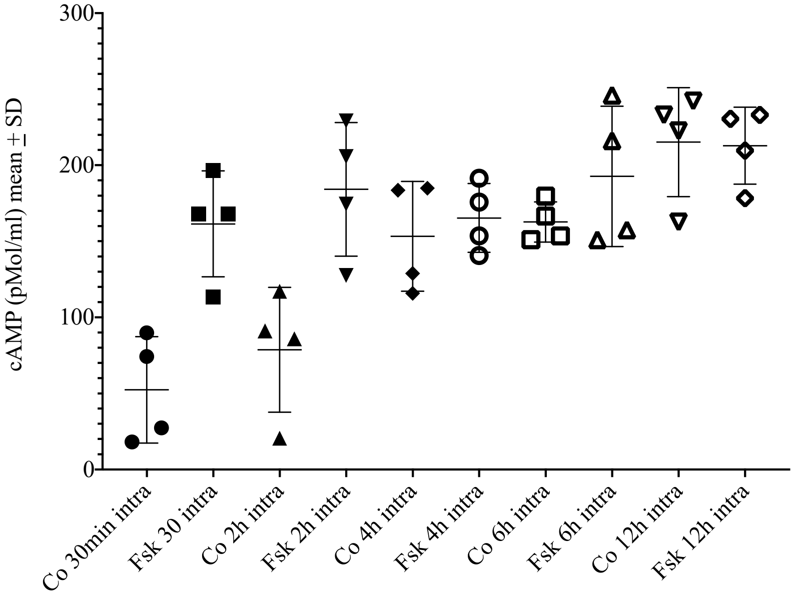

Supplementary Figure S6:
Intracellular cAMP (A) is significantly elevated after 30 minutes and two hours of exposure to forskolin (1µM), but extracellular cAMP (B) is not different at any time measured up to 12 hours.

B

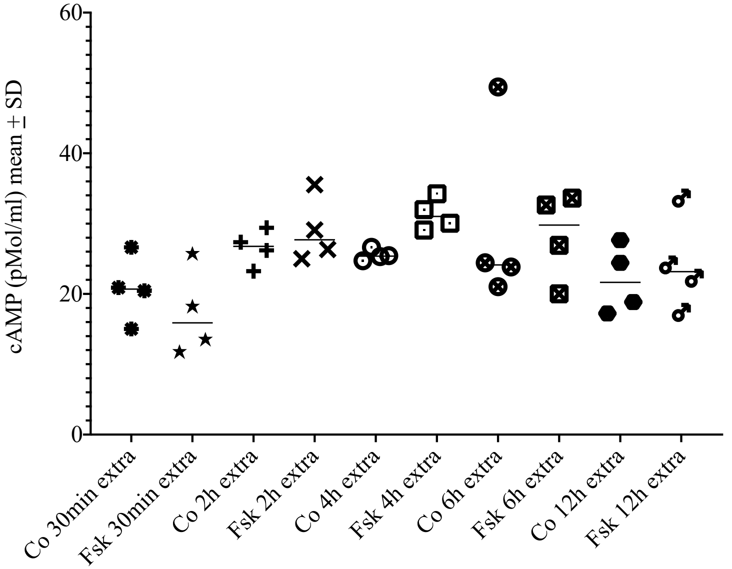

Supplementary Figure S7: The effect of SQ22536 on forskolin-induced CRE-luciferase activity and cell vitality

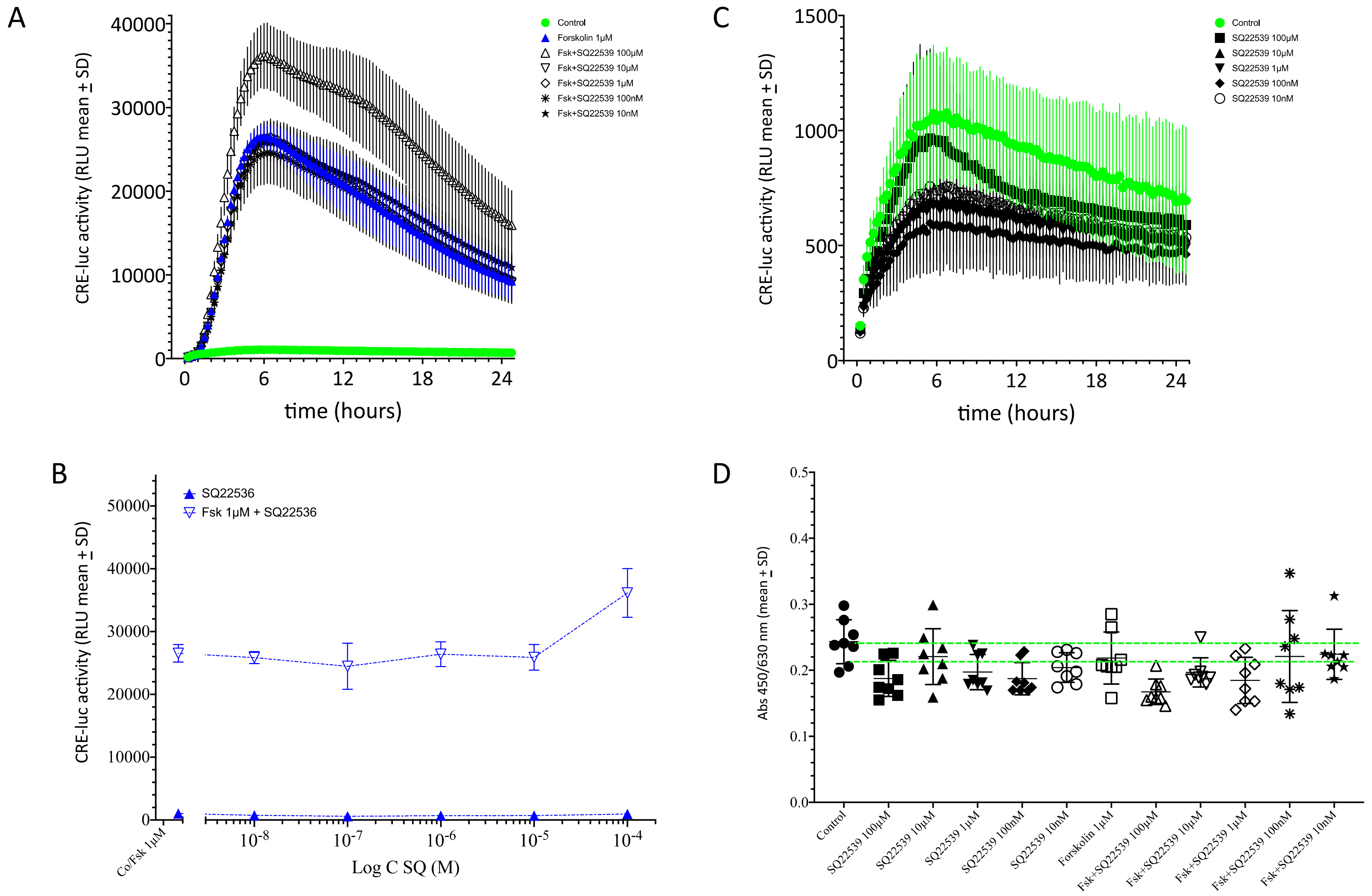

Supplementary Figure S8:
Original Blot images of
the Western blot quantification
in Fig.9B

Co:	1,5,9,13
Fsk 10µM:	2,6,10,14
PMA 1µM:	3,7,11,15
Fsk+PMA:	4,8,12,16

pCREB

ß-actin

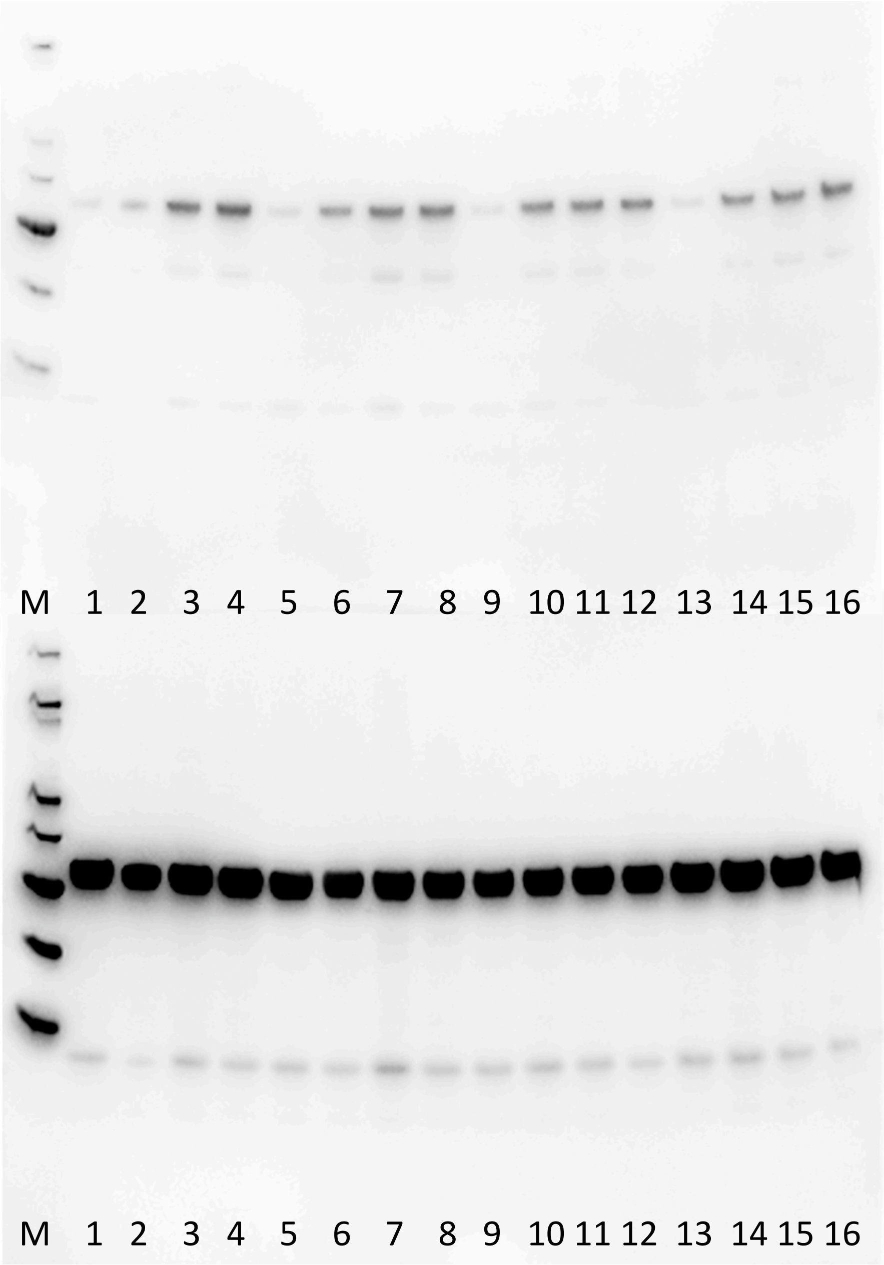

Supplement: Supplementary file 1 [file ijms-23-12226-s001.zip › ijms-1863555-supplementary.pdf]
