# Supplementary material for: Regulation of CRE-Dependent Transcriptional Activity in a Mouse Suprachiasmatic Nucleus Cell Line
Source: Int J Mol Sci. 2022 Oct 13;23(20):12226. doi: 10.3390/ijms232012226 (PMC9602552; doi:10.3390/ijms232012226)
Supplement: Supplementary file 1 [file ijms-23-12226-s001.zip › ijms-1863555-Table S1 Antibodies.pdf]

Table S1. Antibodies.

Antigen	Host	Method	Dilution	Supplier	Notes
AVP	Mouse	IF	1:500	Dr. Hal Gainer, NIH	PS41
VIP	Rabbit	IF	1:500	ImmunoStar, Inc.	20077
VIP	Rabbit	WB	1:2000	ImmunoStar, Inc.	Lot112001
PKA-RI	Rabbit	WB	1:2000	BD-TL	610105
PKA-RIIalpha	Mouse	WB	1:5000	BD-TL	612242
PKA-RIIbeta	Mouse	WB	1:5000	BD-TL	610625
pPKA-S	Rabbit	WB	1:5000	CST	100G7E
pCREB	Rabbit	WB	1:1000	CST	89G3
beta-Actin	Mouse	WB	1:20.000	Sigma	AC-74
betaIIITub	Rabbit	WB	1:1000	CST	TU-20
AKAP220	Rabbit	WB	1:1000	BD-TL	610704
MAP2	Mouse	WB	1:5000	BD-TL	610460
Nestin	Mouse	WB	1:1000	CST	MAB353
GFAP	Mouse	WB	1:1000	CST	Clone
Rabbit IgG	Goat	WB	1:50.000	Santa Cruz	HRP-conj.
Mouse IgG	Goat	WB	1:50.000	DAKO	HRP-conj.

Antigen	Host	Method	Supplier	Dilution	
**pCREB**	Rabbit	Western Blot	Sigma C9102	1:1000	
**pCREB**	Rabbit	Western Blot	SAB 11052	1:1000	
**pCREB**	Rabbit	Western Blot	CST#9198s	1:1000	
**pCREB**	Rabbit	Western Blot	CST#9191s	1:1000	
**pCREB**	Rabbit	Western Blot	87G3; CST	1:1000	
**pCREB**	Rabbit	Western Blot	Millipore 06-519	1:1000	Lot:1924367
**pCREB**	Rabbit	Western Blot	Millipore 06-519	1:1000	Lot:2325090
**PKA RIIα**	Mouse	Western Blot	BD Transduction labs	1:5000	
**pCREB**	Rabbit	Western Blot	R&DAF2510	1:1000	
**p-PKA substrate**	Rabbit	Western Blot	100G7E; CST	1:2000	
